# Health-related quality of life (EQ-5D) after revision arthroplasty following periprosthetic femoral fractures Vancouver B2 and B3 in geriatric trauma patients

**DOI:** 10.1007/s00402-024-05287-5

**Published:** 2024-03-30

**Authors:** Melina Pavlović, Christopher Bliemel, Vanessa Ketter, Julia Lenz, Steffen Ruchholtz, Daphne Eschbach

**Affiliations:** 1 Center for Orthopedics and Trauma Surgery, University Hospital Gießen and Marburg, Philipps - Universität Marburg, 35043 Marburg, Germany; 2 MVZ OCP Kassel, 34123 Kassel, Germany

**Keywords:** Periprosthetic fracture, Vancouver B2, Vancouver B3, Geriatric trauma, Revision arthroplasty, Quality of life

## Abstract

**Introduction:**

The aim of this study was to determine the outcome parameters of revision arthroplasties for periprosthetic femoral fractures (PPFF) with a particular attention to quality of life (QoL) and mobility.

**Materials and methods:**

Retrospective single-center study of PPFF with loose implants that underwent revision arthroplasty. Depending on individual patient characteristics, either an uncemented or cemented revision stem was chosen. Data collection included demographics, complications, clinical course and outcome parameters. Follow-up took place at least one year postoperatively.

**Results:**

Between 2008 and 2016, 43 patients could be included. Most patients (63%) were able to walk independently or with a walking aid after one year and amongst the surveyed patients 77% were able to reside at home. Concerning the QoL assessment, a high index of 0.8 ± 0.1 has been reached after one year. Mortality pointed out to be 9% after one year and 28% in general.

**Conclusion:**

The treatment of PPFF remains challenging. Although complication rates and mortality are high in this frail collective of geriatric patients, revision arthroplasty leads to good postoperative results regarding mobility and quality of life.

**Supplementary Information:**

The online version contains supplementary material available at 10.1007/s00402-024-05287-5.

## Introduction

Projections for different countries are forecasting a higher total number of total hip arthroplasties (THA) over the next decades rising between 12% and 219% [1–3]. The cumulative risk for sustaining a periprosthetic femoral fracture (PPFF) after primary THA has been calculated to be 3.5% within 20 years [4]. According to the projections, an increase of PPFF has to be expected as well. Based on data of different registries, a 4.6% rise in PPFF every ten years has been forecasted for the U.S. over the next 30 years [5].

Risk factors for obtaining a periprosthetic fracture are well described in literature including age > 65 years, female sex, neurological disorders (causing an increased risk of falling), osteoporosis and further diseases that impair bone density such as renal insufficiency and heart failure, small stems, uncemented implantation technique and false drilling holes [4, 6].

PPFF around total hip arthroplasty are commonly classified by the Vancouver classification [7]. According to literature, Vancouver A fractures can usually be treated non-operatively while the other entities undergo operative treatment [8].

Since PPFF have been subject to several investigations over the last years, much is known about individual outcome parameters such as fracture-union, revision rates, perioperative complications and mortality [9].

The reoperation rates after open reduction internal fixation (ORIF) and revision arthroplasty are as high as 23% [10] and more recently 13.7% [6]. Likewise, mortality rates for one-year are 13.4% [6].

Nonetheless, little is known about the quality of life (QoL) and mobility after sustaining a PPFF. The fracture, operation and postoperative course and particularly in combination significantly impact not only the health and mobility but especially the autonomy and QoL of older people. Both are increasingly relevant outcome parameters especially for geriatric patients [11].

Thus, the aim of the present investigation is to analyze in-hospital complications, reoperations and mortality, as well as quality of life, return to pre-fracture mobility and living-conditions in a comparatively large, homogeneous collective of geriatric trauma patients undergoing revision arthroplasty following Vancouver B2 and B3 fractures.

## Materials and methods

Retrospective single-center observation study of patients classified with Vancouver B2 or B3 fractures [7, 12], who underwent revision arthroplasty from 2009 to 2016. Fractures were labeled as interprosthetic fractures if an additional knee arthroplasty was present. Exclusion criteria were pathological fractures and critically injured patients. Ethical approval was given by local authorities (AZ 68/16) and informed consent was obtained from patients or their legal representatives prior to follow-up examination.

For all patients with good bone quality and acceptable distal bone stock a diaphyseal fitting modular uncemented stem was chosen (MRP-TITAN®, Peter Brehm GmbH, Weisendorf, Germany). Whenever there was less than 4 cm of distal bone stock to sufficiently anchor the prosthesis press-fit or in case of poor bone quality, a cemented long stem prosthesis was chosen initially (Cortina, implantcast GmbH, Buxtehude, Germany). No allografts have been used. All patients with cemented revision arthroplasty were allowed full weight bearing postoperatively. In patients with uncemented implants, full or partial loading was allowed depending on the bone stock and the patient’s body weight and abilities.

We assessed the following baseline parameters: age, sex, pre-fracture mobility and inpatient treatment data (medication, comorbidities, length of surgery) from existing case notes. The age-adjusted CCI [13] has been calculated accordingly. Type of admission, discharge and length of in hospital stay was documented in the hospital information system. Postoperative outcome measures were divided into “minor complications” and “major complications” depending on their need for surgical revision. Minor complications included urinary tract infection, respiratory complications, impaired wound healing, thrombosis or decubitus. Major complications with reoperation were precisely gathered regarding time and fashion of the procedure.

Follow-ups took place at least one year after the revision surgery had been performed and were executed by an experienced physician and a study nurse. Patients who were able to come or could be taken to the hospital underwent clinical examination, the others were interviewed via telephone. During follow-up, we assessed the quality of life by use of the EQ-5D-5L. This measurement tool focuses on different categories of physical and mental health as well as autonomy (Mobility, Anxiety / Depression, Usual activities, Self-care, Pain / Discomfort) and uses three levels of response (1: no problems; 2: mild/moderate problems; 3: severe problems). Valuations have been conducted for several countries, including Germany. The EQ-5D-5L index was calculated using the crosswalk value set for Germany [14]. The HHS was obtained during clinical examination. Furthermore, the modified HHS was calculated as well since we could not perform a clinical examination in all patients. The current living situation and mobility have been inquired about as well. Concerning the postoperative mobility, the last observation from the regular postoperative visits that has been documented in the case files has been carried forward in case of a drop-out.

Regular radiological follow-ups were performed during the first postoperative week and after 6 weeks. All patients who agreed were subsequently discharged to a rehabilitation ward or a geriatric aftercare unit.

Data collection and pseudonymisation was performed using an Excel 2020 database (Microsoft® Corporation, Redmond, WA, USA). IBM® SPSS® Statistics 27.0 (Statistical Package for the Social Science, IBM Corporation, Armonk, NY, USA) was used for statistical analysis. Descriptive statistics were used to describe clinical characteristics, complications and outcomes. Data were presented as means, standard deviations, and frequencies. The Kruskal-Wallis and Mann-Whitney-U tests were used to compare means. A *p*-value of < 0.05 was considered significant.

## Results

### Baseline parameters

We were able to include 43 patients who met the inclusion criteria mentioned above from 2009 to 2016. The acquired baseline data were collected from all those patients. All subgroups have been tested for homogeneity. Analysis demonstrates that the groups show the same distribution of age and gender. Concerning CCI, the post-hoc test revealed a difference between Vancouver B3 and interprosthetic fractures (*p*-value = 0.028) with sicker patients in the Vancouver B3 group. All other comparisons (Vancouver B2 – B3, Vancouver B2 – Interprosthetic) did not show any differences (*p*-values 0.181 and 0.643).

Nine patients were lost to follow-up and 12 patients died before the follow-up took place. The remaining 22 patients agreed to a follow-up. Of these, 21 patients were clinically re-examined, and one patient answered a telephone interview due to a home-bound situation. The average follow-up time in this cohort was 48 ± 33 months with a minimum of one year.

Baseline data are shown in Table 1.

**Table 1:** Baseline characteristics, descriptive analysis in total and divided into subgroups according to fracture pattern

	Total	Vancouver B2	Vancouver B3	Interprosthetic	p-value
Number	**43**	56% (*n* = 24)	30% (*n* = 13)	14% (*n* = 6)	
Age	**78 ± 9.2**	78 ± 8.5	81 ± 7.3	72 ± 9.6	*0.118*
Sex (% female)	**67%**	63%	80%	67%	*0.646*
CCI	**5.2 ± 1.7**	5.2 ± 1.5	5.9 ± 2.1	4.0 ± 0.6	0.034*

(CCI: age-adjusted Charlson comorbidity index, significance level *p* < 0.05)

### Operative treatment

All patients underwent a revision arthroplasty with either a modular diaphyseal fitting stem (74%, *n* = 32) or a long cemented monobloc stem (26%, *n* = 11). The mean duration of all procedures was 149 ± 35 min with 150 ± 34 min in the modular stem group and 145 ± 40 min in the long non-modular cemented stem group. The difference was not statistically significant neither about the implant (*p* = 0,445) nor the initial fracture (*p* = 0,974).

### Inpatient treatment

The inpatient treatment lasted 16 ± 8 days on average. 39 of the patients required a short monitoring interval on the intensive care unit (ICU). 11 of these patients needed a surveillance longer than 24 h (2.4 ± 1.5 days on average). A total of 36 (84%) patients were subject to transfusion. A cumulative need for substitution throughout the whole hospitalization period has been calculated for all patients with 3.1 ± 2.8 red cell concentrates.

### Complications

Regarding nonsurgical complications, a total of 17 patients (39%) suffered from either a urinary tract infection (*n* = 6), respiratory complications (*n* = 3), impaired wound healing (*n* = 4), thrombosis (*n* = 2) or decubitus (*n* = 2).

A surgical revision due to postoperative complications had to be performed in 10 (23%) cases. A detailed overview is shown in Table 2.

**Table 2:** Surgical complications

Complication	n (%)	Time (d)	Procedure
Deep infection Deep infection Deep infection	3 (7%)	9 36 58	Rinsing, exchange of mobile components Multiple revisions, two-staged complete exchange Rinsing, exchange of mobile components + vacuum therapy
Superficial infection Superficial infection Superficial infection	3 (7%)	9 15 25	Rinsing Rinsing Rinsing and vacuum therapy + establishment of fistula
Dislocation	1 (2%)	10	Revision of hip component (Dual mobility acetabular cup, Avantage$\text{\circledR },$Biomet)
Sewed-on drain	1 (2%)	3	Removal of drain
Hematoma Hematoma	2 (5%)	4 14	Rinsing Rinsing

n = Number of patients who underwent revision; Time = postoperative timespan when first revision occurred, d = days; Procedure = Revisions performed per patient

It must be mentioned that 3 cases were simple procedures, like the removal of a hematoma or the extraction of a sewed-on drain. Only 16% (*n* = 7) of cases resulted in major operative revision with an exchange of all or single components or proven periprosthetic joint infection (PJI).

### Living conditions

31 of the 41 patients who could be discharged (72% of all patients), went to a specific geriatric rehabilitation (*n* = 24) or a subsequent trauma rehabilitation (*n* = 7). Nine patients (21%) were discharged home. Only one patient, who has initially been admitted from a nursing home, directly went back to that institution without rehabilitation. Amongst the patients of whom data could be obtained concerning their living status, 21 (49% of all patients) were living at home, either with their spouse, their relatives or alone. The remaining 2 (5% of all patients) were living in a nursing home.

### Pre- and postop mobility

Concerning the postoperative mobility, seven patients were mobile without any aid and 20 patients were mobile with a walking aid (walking frame or cane) during follow-up. Two of the included patients were in need of a wheelchair and one was bedridden at the time of follow-up. Summarized, 90% of the patients of whom data could be collected, were mobile (63% of all patients).

The HHS was 70 ± 29 on average, displaying only a fair functional outcome but with a severe standard deviation. Having a closer look at the HHS, we identified the most variable items to be limping (3.4 ± 4.2 of 11 points max), walking distance (4.5 ± 3.9 of 11 points max) and the need for walking aids (3.8 ± 4.6 of 11 points max).

### Quality of life

The analysis of the quality of life assessment (EQ-5D-5L) showed that 13 of the 22 examined patients stated no restrictions at all concerning the subitem of mobility (30% of all patients). The detailed results can be found in Table 3 below. The mean index value was 0.8 ± 0.1.

**Table 3:** Distribution of answers (EQ-5D-5L, EuroQuol Health Questionnaire 5D) of the 22 patients during follow-up

	Mobility		Self-care	Usual activities	Pain	Anxiety
**1 No problems**		13	11	9	8	12
**2 Moderate problems**		8	7	8	11	10
**3 Severe problems**		1	4	5	3	0

### Mortality

**Fig. 1:**
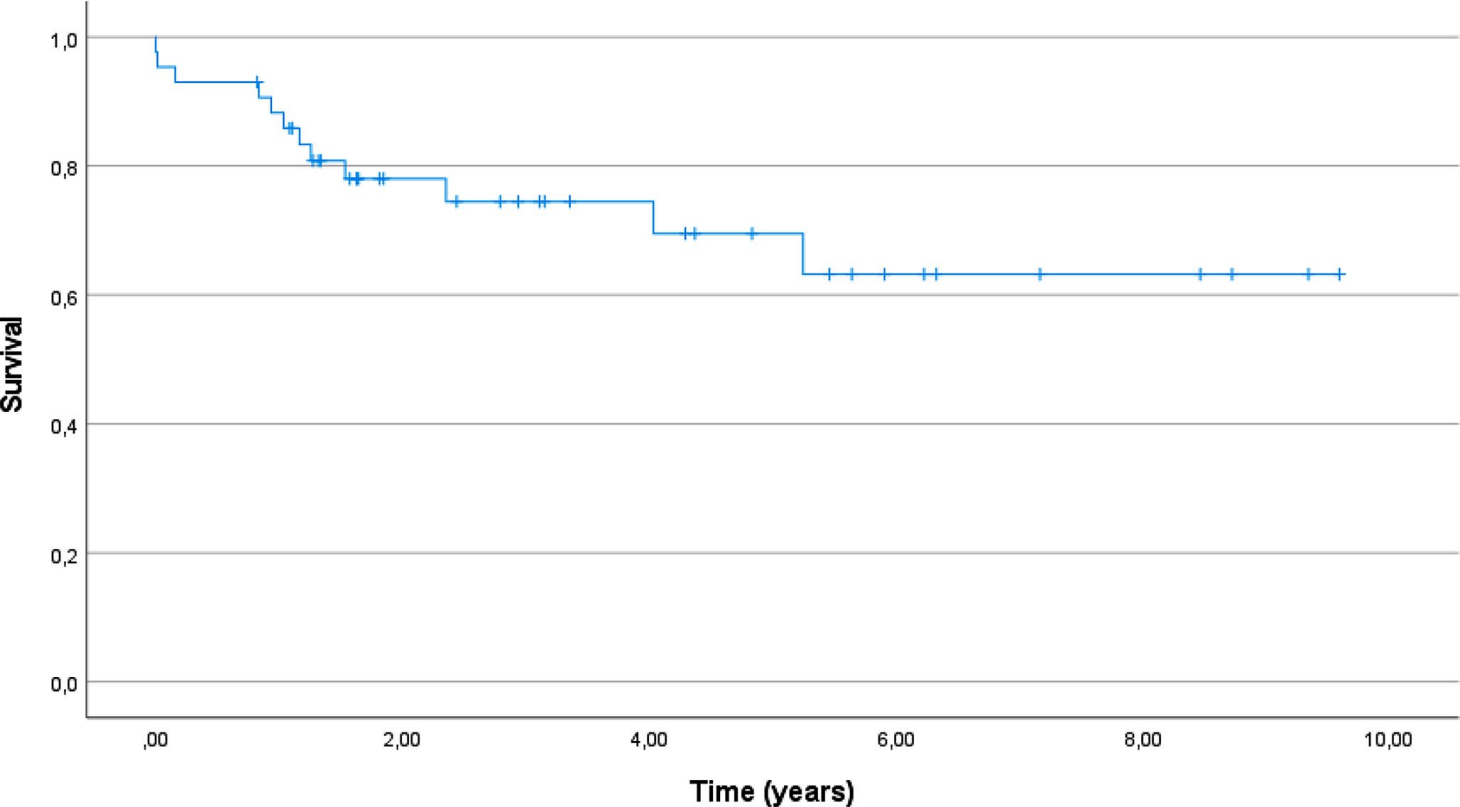
Kaplan Meier Curve, survival rate during the study period

Within the first postoperative year retrospective analysis showed that 4 patients had died, leading to a one-year mortality rate of 9%. Two of those deaths happened during inpatient treatment. The first patient died from cardiovascular failure after a bone cement reaction. The second patient died as well from cardiovascular failure, but after a cementless arthroplasty because of a pulmonary embolism. The other two remaining deaths happened after dismissal and were due to comorbidities. Over the study period, 12 patients died, leading to an overall mortality of 28%. The calculated Kaplan Meier curve for survival can be seen in Fig. 1.

## Discussion

**Fig. 2:**
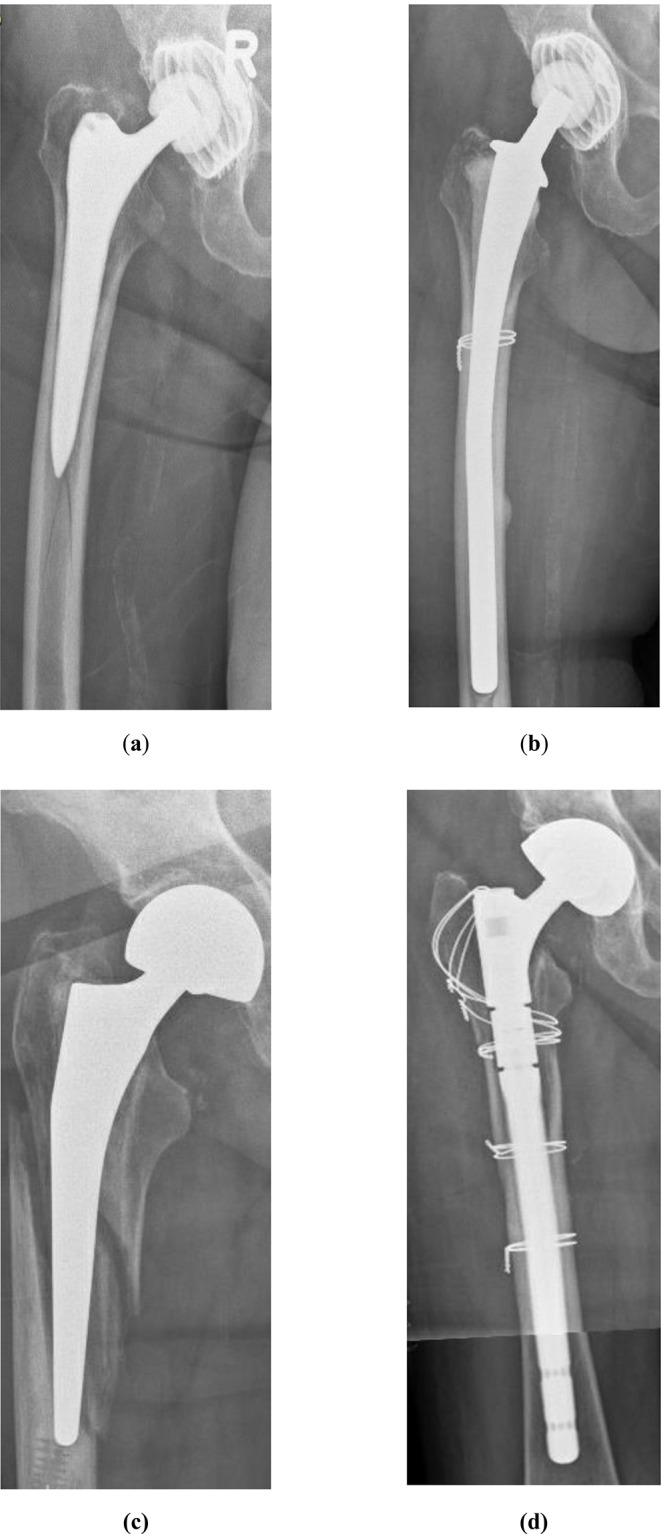
**(a)** Vancouver B2 fracture, **(b)** postoperative imaging after implantation of a long-cemented stem; **(c)** Vancouver B3 fracture, **(d)** postoperative imaging after implantation of a modular uncemented stem

This retrospective single-center observation study analyzed the clinical outcome of patients that underwent revision arthroplasty due to PPFF classified as Vancouver B2 or B3 fractures (Fig. 2). The main study results are that most patients regained their ability to walk and their ability to reside at home. Although the one-year mortality rate wasn’t negligible at approximately 9%, the EQ-5D-5L assessment revealed high scores concerning the health-related quality of life among the survivors.

The knowledge about treatment and outcome of PPFFs is rapidly increasing over the last years, substantive information about the postoperative course is still rare. Due to the high age, immobility and comorbidities of the geriatric collective, the maintenance of follow-up is extraordinary difficult. Thus, the study’s drop-out rate of 48% (*n* = 21) in total, attributable to deaths and drop-outs.

As mentioned above in the introduction, age and female sex are commonly described patient-specific risk factors even though data remains inconclusive to date [4, 15, 16]. In our collective we documented a mean age of 78 ± 9.2 at the time the revision arthroplasty took place with a clear accumulation of female patients (67%), supporting the preexisting data.

An overall reoperation rate of 18.6% in 1381 surgically treated Vancouver B fractures of all sub-types has recently been described by Chatziagorou et al. [17]. In a review of 22 studies, Khan et al. demonstrated a similar reoperation rate of 12,4% for Vancouver B2 fractures und 14,4% Vancouver B3 fractures [16]. In our collective, a surgical procedure due to postoperative complications had to be performed in 10 (23%) of the cases, 7 (16%) with a major revision (PJI and/or exchange of components). One plausible reason for our comparatively high reoperation rate is our aggressive strategy for irrigating wounds with hematoma or evidence of superficial wound infection. We hope to be able to avoid deep infections with disastrous outcomes in the course. Consequently, only one patient had to undergo a two-staged complete exchange due to a PJI.

76% of our patients could be relocated to a subsequent cure and gained from a specific (geriatric) rehabilitation. At the time of data acquisition, 49% (*n* = 21) of all patients were living at home, either with their spouse, their relatives or alone. The remaining patients were living in a nursing home. These data show that many patients, even of older age and after sustaining a complex surgery, could be led back home in familiar surroundings which most certainly helped their personal perception of regained autonomy and quality of life and is supposed to be one the most important outcome parameters for geriatric patients [11].

Regarding mobility, of the 30 patients, of whom information could be obtained, seven were mobile without any device and another 20 with walking frame or cane. Totaled up, 35% of all patients gained back their preoperative status concerning the need for walking aids and walking distance. Two of those patients even improved their mobility compared to their preoperative status, probably because of a symptomatic instability or implant loosening prior to PPFF. Mulay et al. published a prospective study of 22 patients in 2005. In this study distinct information about the postoperative mobility has been given. According to the authors, likewise to our own results, none of the patients was immobile. Only one patient, who has been bedridden before sustaining the PPFF, was not able to walk after the revision arthroplasty [18].

Nevertheless, it must be mentioned, that HHS was 70 ± 29 on average showing only fair results. The most deduction in this score was due to walking aids, walking distance and limping. In the quoted study of Mulay et al., the HHS after the one-year follow-up equally was only 69 [18]. Moreta et al. displayed their own results as well as several other studies with an HHS within a range of 67–83. The reported follow-up period thus was considerably longer than our own (1–8.3 years) [19]. For these geriatric patients, we believe, the fair result of the HHS does not sufficiently describe the level of mobility and satisfaction the older patient subjectively experiences.

The findings of our study are supporting this thesis. Concerning quality of life (EQ-5D-5L), we could show that 59% of the surveyed patients stated no restrictions at all concerning the subitem of mobility (30% of all 43 patients). It shows that the perception of restriction is often situation- and need-dependent and can only inadequately be gathered in scores, a factor that has been reported in some publications on self-assessment of functional status as well [20]. The mean index value of the EQ-5D-5L in our study was 0.8 ± 0.1. Buecking et al. also used this measurement tool in a collective of geriatric trauma patients following a proximal femoral fracture. They could show a pre-fracture EQ-5D-5L of 0.7 ± 0.3 for slightly older (81 years vs. 78 years) yet healthier patients (CCI 2.4 vs. 5.2 in our study), indicating that the patients who participated in our follow-up examination even reached a better health-related QoL postoperatively than an uninjured geriatric collective. It must be mentioned though, that the prefracture assessment was performed retrospectively from memory after admission in that study. Unfortunately, the follow-up EQ-5D-5L values have only been reported upon dismissal but not after one year in the above mentioned study. Within this short interval, a dramatic deterioration to 0.5 ± 0.3 has been seen [21]. Most studies did not assess health-related QoL after an adequate period of one year but only shorter postoperative intervals [11]. Ekström et al. however illustrated an EQ-5D index of 0.6 one year after trochanteric fracture and of 0.5 after subtrochanteric fracture [22, 23]. Those comparisons suggest that our patients reached a veritable level of their quality of life after one year, even better than a comparable group of patients with proximal femur fractures.

The mortality after PPFF in general has been stated up to 13% after one year [6]. Our own data showed an 9% mortality rate for the first postoperative year with an overall mortality of 28% for the whole follow-up period of 48 ± 33 months. The mortality after one year has therefore been shown to be comparable than the mortality rates after proximal femoral fractures [24]. With 4.6%, the in-hospital mortality rate in our collective also corresponds to the in-hospital mortality rate for hip fractures with 6.2% [21].

Our study has some limitations. First, the retrospective design of this investigation shows known limitations, including the fact that the follow-up period shows a high variance from one year to 9.6 years maximum. Further, the follow-up examination could only be performed for 22 patients. Even though patients’ records were carefully reviewed, data is strongly dependent on documentation quality. In addition, due to a lack of prefracture values for EQ-5D-5L, we could not identify a progression of that value but only a singular quality.

## Conclusions

Our data show a substantial number of patients were able to gain back their preoperative mobility and living status after revision arthroplasty due to PPFF. Despite the geriatric group and complexity of the operation, the patients regained a sufficient level of their autonomy and QoL. Further investigations concerning influencing factors and a prospective study design is most certainly needed.

### Electronic supplementary material

Below is the link to the electronic supplementary material.

Supplementary Material 1
