# Supplementary material for: Health-related quality of life (EQ-5D) after revision arthroplasty following periprosthetic femoral fractures Vancouver B2 and B3 in geriatric trauma patients
Source: Arch Orthop Trauma Surg. 2024 Mar 30;144(5):2141–8. doi: 10.1007/s00402-024-05287-5 (PMC11093848; doi:10.1007/s00402-024-05287-5)
Supplement: Supplementary file 1 — Supplementary Material 1 [file 402_2024_5287_MOESM1_ESM.docx]

Melina Pavlović^1*^, Christopher Bliemel^1^, Vanessa Ketter^1^, Julia Lenz^1^, Steffen Ruchholtz^1^, Daphne Eschbach^2^

Health-related quality of life (EQ5D) after revision arthroplasty following periprosthetic femoral fractures Vancouver B2 and B3 in geriatric trauma patients

**Conflicts of Interest:** The authors declare no conflict of interest.
